# The OsmiR396c‐OsGRF4‐OsGIF1 regulatory module determines grain size and yield in rice

**DOI:** 10.1111/pbi.12569

**Published:** 2016-05-17

**Authors:** Shuangcheng Li, Fengyan Gao, Kailong Xie, Xiuhong Zeng, Ye Cao, Jing Zeng, Zhongshan He, Yun Ren, Wenbo Li, Qiming Deng, Shiquan Wang, Aiping Zheng, Jun Zhu, Huainian Liu, Lingxia Wang, Ping Li

**Affiliations:** ^1^ Rice Research Institute Sichuan Agricultural University Wenjiang Sichuan China; ^2^ State Key Laboratory of Hybrid Rice Sichuan Agricultural University Chengdu China

**Keywords:** rice, grain size, growth‐regulating factor, miR396, GRF‐interacting factors

## Abstract

Grain weight is the most important component of rice yield and is mainly determined by grain size, which is generally controlled by quantitative trait loci (QTLs). Although numerous QTLs that regulate grain weight have been identified, the genetic network that controls grain size remains unclear. Herein, we report the cloning and functional analysis of a dominant QTL,* grain length and width 2* (*GLW2*), which positively regulates grain weight by simultaneously increasing grain length and width. The *GLW2* locus encodes OsGRF4 (growth‐regulating factor 4) and is regulated by the microRNA miR396c *in vivo*. The mutation in *OsGRF4* perturbs the OsmiR396 target regulation of *OsGRF4*, generating a larger grain size and enhanced grain yield. We also demonstrate that OsGIF1 (GRF‐interacting factors 1) directly interacts with OsGRF4, and increasing its expression improves grain size. Our results suggest that the miR396c‐OsGRF4‐OsGIF1 regulatory module plays an important role in grain size determination and holds implications for rice yield improvement.

## Introduction

Grain weight, which is mainly determined by grain size (length, width and thickness), is a highly important component of rice yield. Grain size is predominantly controlled by quantitative trait loci (QTLs). Recently, several QTLs regulating grain size have been molecularly identified and functionally analysed, providing useful information for understanding their mechanisms and use in rice breeding (Zuo and Li, 2014). A number of studies have identified QTLs that negatively regulate the grain size in rice, such as *GW2* (Song *et al*., 2007), *GW5/qSW5* (Shomura *et al*., 2008; Weng *et al*., 2008), *GS3* (Fan *et al*., 2006; Mao *et al*., 2010), *TGW6* (Ishimaru *et al*., 2013) and *GL3.1/qGL3* (Qi *et al*., 2012; Zhang *et al*., 2012). Recently, several positive regulators have been well characterized. Amongst these regulators, *GS5* encodes a putative serine carboxypeptidase (Li *et al*., 2011), *GW8* encodes OsSPL16 (Wang *et al*., 2012), *GW6a* encodes a new‐type GNAT‐like protein (Song *et al*., 2015) and *GW7/GL7* encodes a TONNEAU1‐recruiting motif protein (Wang *et al*., 2015a,b). Most recently, *GS2* has been reported to positively regulate both grain width and length by promoting cell division and cell expansion (Hu *et al*., 2015). Interestingly, overexpression of rice OsmiR397 enlarges grain size and promotes panicle branching by down‐regulating OsLAC, a laccase‐like protein, which shows a regulatory role of miRNA in controlling rice seed size (Zhang *et al*., 2013). Although numerous QTLs regulating grain weight have been currently identified, the understanding of the precise mechanisms remains elusive.

Growth‐regulating factors (GRFs) are plant‐specific transcription factors (Omidbakhshfard *et al*., 2015) that were firstly identified for their roles in stem and leaf development (Horiguchi *et al*., 2005; Kim and Kende, 2004; Kim *et al*., 2003; van der Knaap *et al*., 2000). However, emerging data have demonstrated such transcription factors to be also important for other various developmental processes, such as root development (Bazin *et al*., 2013; Hewezi *et al*., 2012; Rodriguez *et al*., 2015; Vercruyssen *et al*., 2011), floral organ development (Liang *et al*., 2014; Liu *et al*., 2014), seed formation (Hu *et al*., 2015; Liu *et al*., 2012), longevity (Debernardi *et al*., 2014) and stress response (Casadevall *et al*., 2013; Hewezi *et al*., 2012; Kim *et al*., 2012).

A total of nine GRF members exist in *Arabidopsis thaliana* (Omidbakhshfard *et al*., 2015), and twelve exist in rice (Choi *et al*., 2004a; Omidbakhshfard *et al*., 2015). The majority of plant GRFs consist of two conserved domains: the QLQ domain, which is considered important for protein–protein interactions, and the WRC domains, which is expected to be involved in DNA binding (Choi *et al*., 2004b; Kim *et al*., 2003; van der Knaap *et al*., 2000). These two classically conserved domains suggest at least two important aspects of GRF molecular functions in its signal network. These aspects include protein–protein interactions with its partner and direct DNA binding of the downstream target for expression regulation (Choi *et al*., 2004a; Omidbakhshfard *et al*., 2015). GRF‐interacting factors (GIFs) are the major partners of GRFs through their conserved QLQ domains (Horiguchi *et al*., 2005; Kim and Kende, 2004). GIF1 has been mainly reported to participate in cell proliferation control during leaf development by interacting with GRFs (Horiguchi *et al*., 2005; Kim and Kende, 2004). GIF1 also functions in adaxial/abaxial patterning (Horiguchi *et al*., 2011; Iwakawa *et al*., 2007; Xu *et al*., 2003), establishment of cotyledon identity (Kanei *et al*., 2012) and chromatin remodelling by the interactions to other proteins (Debernardi *et al*., 2014; Vercruyssen *et al*., 2014). In contrast to the protein–protein interactions of GRFs, research that focuses on the downstream target of GRFs is currently limited. Only few target genes, such as the *KNOX* gene (Kuijt *et al*., 2014; Osnato *et al*., 2010), or *OsCR4* and *OsJMJ706* (Liu *et al*., 2014), were identified.

Another important mechanism underlying GRF function is the extensive post‐transcriptional control of *GRF* by microRNA396, an ancient miRNA family (Omidbakhshfard *et al*., 2015). Most, but not all, *GRFs* are miR396 targets. MiR396 directly targets *GRF* transcripts, thereby negatively regulating the latter's expression levels. Several reports, mostly in *A. thaliana*, have established a miR396‐*GRF* regulatory module, which operates in various developmental processes, such as stem/leaf development (Das Gupta and Nath, 2015; Debernardi *et al*., 2014; Liu *et al*., 2009; Mecchia *et al*., 2013; Rodriguez *et al*., 2010; Wang *et al*., 2010), root development (Bazin *et al*., 2013; Hewezi *et al*., 2012; Rodriguez *et al*., 2015), reproductive organ development (Baucher *et al*., 2013; Liang *et al*., 2014; Liu *et al*., 2014) and environmental response (Casadevall *et al*., 2013; Hewezi *et al*., 2012). However, reports of this regulatory module in other plants, especially in more economically important crops, are relatively lacking. Recently, the miR396d–*GRF* regulatory module is reported to be involved in floral organogenesis in rice (Liu *et al*., 2014). Interestingly, *GRFs* may also affect miR396 transcript levels and the expression of other *GRFs* possibly by a reciprocal feedback regulation (Hewezi and Baum, 2012); however, the underlying molecular details are unknown.

Herein, we report the map‐based cloning and functional analysis of a dominant QTL, *GLW2*, which positively regulates grain weight by simultaneously increasing grain length and width. The *GLW2* locus encodes OsGRF4 and is regulated by miRNA OsmiR396 *in vivo*. The mutation in *OsGRF4* perturbs OsmiR396‐directed regulation of *OsGRF4*, generating a larger grain size and enhanced grain yield. We also demonstrate that OsGIF1 directly interacts with OsGRF4, and increasing its expression improves grain size. Our results suggest that the miR396‐OsGRF4‐OsGIF1 regulatory module plays an important role in grain size determination and may help improve rice grain yield in rice.

## Results

### *GLW2* allele from 307R significantly increases rice grain weight by simultaneously regulating grain length and width

**Figure 1:**
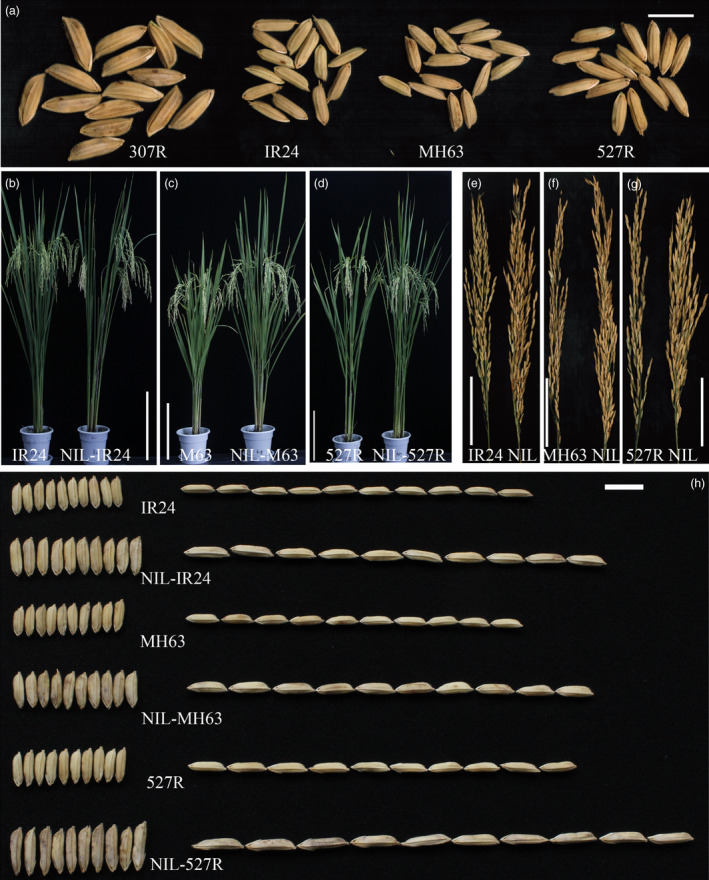
Comparisons between near‐isogenic lines (NILs) and the recurrent parents. (a) Grains of 307R, IR24, MH63 and 527R; scale bar, 10 mm; (b–d) plant comparisons of NILs and the recurrent parents, IR24/NIL‐IR24 (b); MH63/NIL‐MH63 (c); 527R/NIL‐527R (d); scale bar, 30 cm; (e–g) panicle comparisons of NILs and the recurrent parents, IR24/NIL‐IR24 (e); MH63/NIL‐MH63 (f); 527R/NIL‐527R (g); scale bar, 10 cm; (h) grain size phenotype of NILs and the recurrent parents; scale bar, 10 mm.

An extra‐large grain rice line designated as 307R, with a 1000‐grain weight (TGW) of 64 g (Figure 1a), was identified from our breeding materials. To verify the usefulness of this trait, we crossed 307R with three elite rice restorer lines, IR24 (with a TGW of 28 g), MH63 (with a TGW of 30 g) and 527R (with a TGW of 35 g), respectively, which have been widely used as the male parents of commercial hybrid rice (Figure 1a). Three near‐isogenic lines (NILs; NIL‐IR24, NIL‐MH63 and NIL‐527R) were developed from different backgrounds (Figure 1b–d). The grains of NILs were significantly larger than those of the recurrent parents (Figure 1h), showing apparently increased grain length (from 21.42% to 31.69%) (Figure S1b), grain width (from 22.43% to 27.38%) (Figure S1c) and grain weight (from 26.91% to 52.97%) (Figure S1a). As a result, these improvements led to a 14.93%–26.0% increase in grain yield per plant in NILs compared with the recurrent parents (Figure S1e). Moreover, NILs also exhibited a tendency to improve in grain number per panicle, although the effect was not significant (Figure S1d). These results suggest that the allele from 307R can increase rice grain weight by regulating grain length and width. Grain size from heterozygous plants is close to that from the larger homozygous plants; the allele from the 307R is likely an incomplete dominant allele for rice grain size and weight control.

### *GLW2* primarily regulates grain size by promoting cell expansion

The spikelet hull of NIL‐IR24 was apparently larger than that of IR24 both in length and width (Figure S2a). Histological sectioning analysis of the hull indicated that the number of the outer parenchyma cells was significantly increased by 9.4% in NIL‐IR24 compared with IR24 (Figures S2b,c). In addition, scanning electron microscopy of the grain husk revealed that NIL‐IR24 exhibited a significantly enlarged cell volume than that of IR24 (Figure S2d), showing a sharp decrease in epidermal cell numbers of outer glume per unit area (43.12%) (Figure S2e). Consistent with this result, the length and width of epidermal cells of the outer glumes increased by 59.82% and 30.36%, respectively, in NIL‐IR24 compared with those in IR24 (Figures S2f, g). These results suggest that the large grain gene allele of NILs predominantly promotes cell expansion but also increases cell proliferation.

### *GLW2* encodes OsGRF4, a functional transcription factor

Using 180 F_2_ short‐grain individuals generated from a cross of 307R/IR24, we firstly mapped the QTL to chromosome 2 and designated as *GLW2*. The *GLW2* locus was further narrowed down to a 160‐kb interval between the markers H2 and Z4 using 2500 short‐grain individuals generated from a BC_3_F_2_ population of the same cross. Finally, we limited the *GLW2* locus to a 15.3‐kb interval, which contains only one candidate gene *LOC_Os02g47280*, between the markers M2 and M8 by fine genotyping of eight fixed recombinants. Sequence comparison revealed that the coding regions of the *LOC_Os02g47280* gene contained four polymorphisms between *Nipponbare* and 307R. However, only one polymorphism (TC487‐488AA) was conserved between IR24/MH63/527R and their NILs, which suggest that the TC487‐488AA mutation is the causal mutation for the large grain size (Figure S3).

This *LOC_Os02g47280* gene encodes OsGRF4, which is preferentially expressed in young panicles; however, compared with *Nipponbare*, 307R exhibits an obviously elevated level of *OsGRF4* transcripts in most tissues (Figure 4a). This fact suggests that the 2‐bp mutation may lead to an elevated expression of *OsGRF4* and the final large grain in 307R.

**Figure 2:**
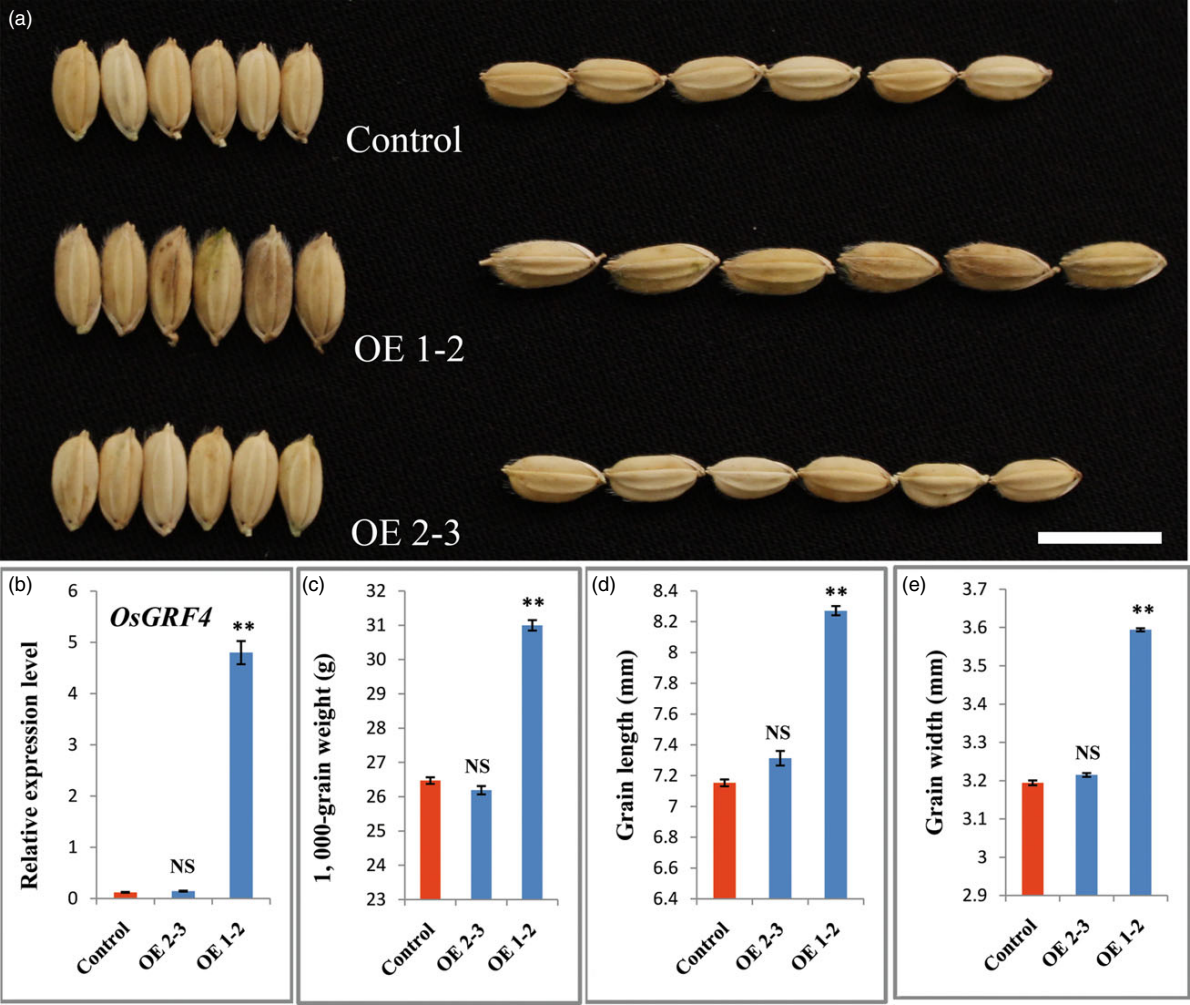
*OsGRF4* overexpression increases rice grain size and weight. (a) Grain comparisons of the control and *OsGRF4* overexpression plants; scale bar, 10 mm; (b) *OsGRF4* expression levels of plants in (a); (c) TGW of plants in (a); (d) grain length of plants in (a); (e) grain width of plants in (a); values are all shown as means ± standard error of the mean (SEM, **P < 0.01.)

**Figure 3:**
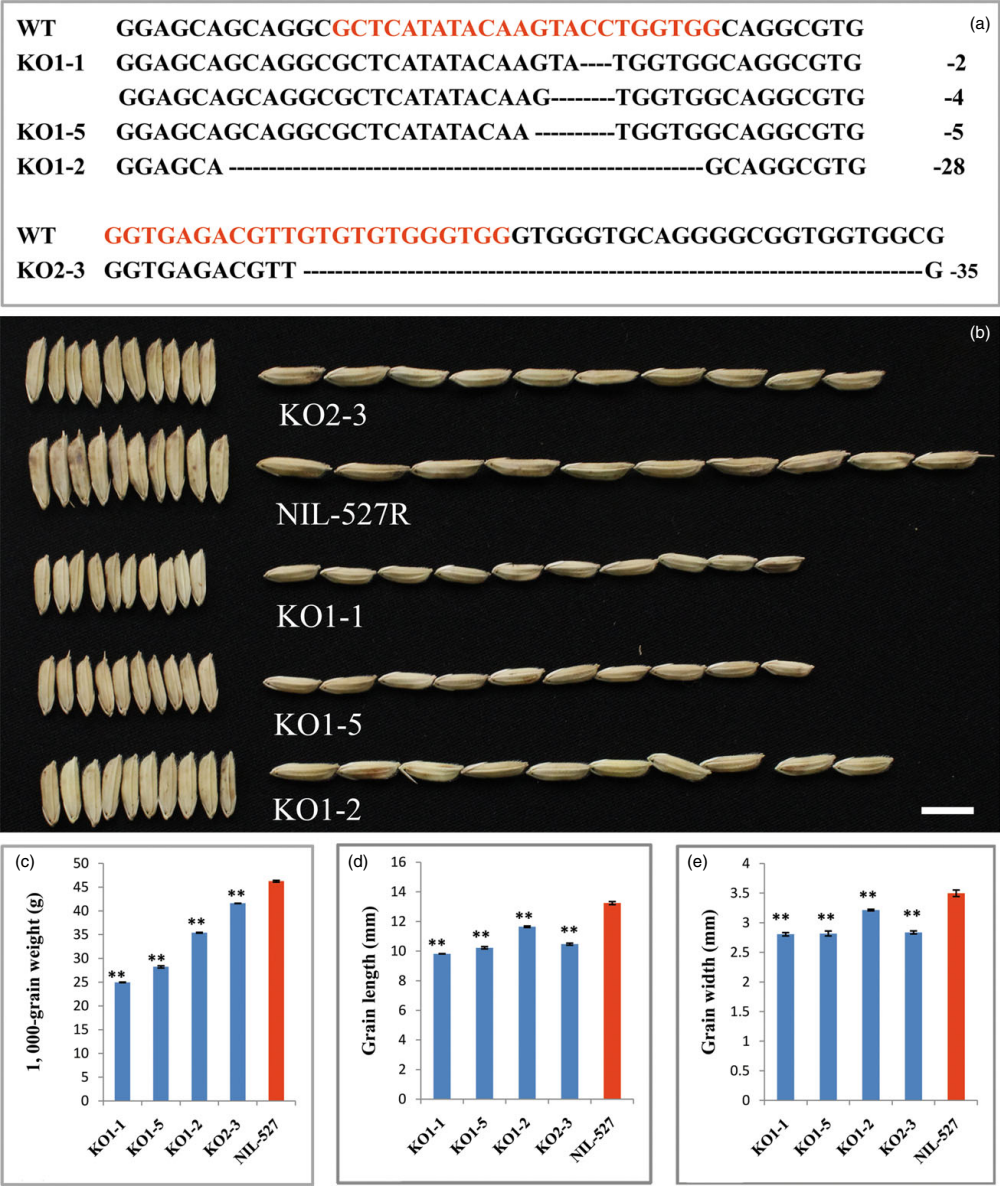
Knock out (KO) of *OsGRF4* in NIL‐527R decreases grain size and weight. (a) Sequence comparisons of the targeting site of the KO plants; the sequence in red is the gRNA target site. Note that KO1‐1 plant is bi‐allelic and others are homozygous. (b) Grain comparisons of the control and *OsGRF4* KO plants; scale bar, 10 mm; (c) KGW of plants in (b); (d) grain length of plants in (b); (e) grain width of plants in (b); values are all shown as means ± SEM (**P < 0.01).

To confirm whether *OsGRF4* corresponds to *GLW2*, we firstly generated an overexpression construct in which the *OsGRF4* from the IR24 background was driven by the 2 × 35S promoter and introduced into *Nipponbare*. Investigation indicated that the transgenic plants were increased apparently in grain size and weight (Figures 2a,c–e). The increased grain size and weight were further confirmed to be a consequence of *OsGRF4* overexpression (Figure 2b). We next generated two gRNA constructs, which were introduced into NIL‐527R to knock out (KO) the *OsGRF4* gene in a CRISPR/CAS9 strategy (Miao *et al*., 2013). Several independent bi‐allelic or homozygous KO plants were obtained (Figures 3a,b) and showed obvious decreases in grain size and weight (Figures 3b–e). These results demonstrate that *OsGRF4* is responsible for the grain size and weight phenotype and its elevated expression benefit to a large and heavy grain.

The transient expression of a GLW2–YFP (yellow fluorescent protein) fusion protein in rice protoplasts showed that GLW2–YFP localized mainly to the nucleus (Figure S4a), which is consistent with the finding that OsGRF4 is a plant‐specific transcription factor (Omidbakhshfard *et al*., 2015). We then demonstrated that OsGRF4 holds a transcription activation activity, and the activation domain was located in its C‐terminal region as demonstrated by a series of truncation analyses in yeast cells (Figure S4b).

### *GLW2* is directly regulated by OsmiR396c

**Figure 4:**
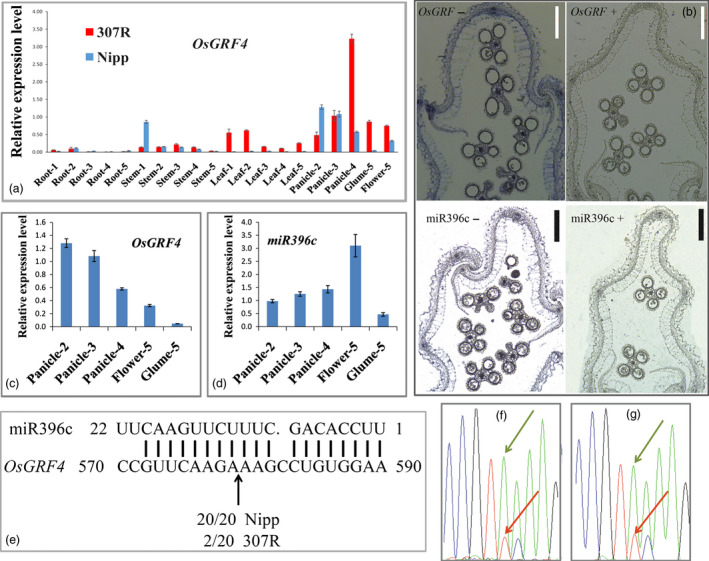
OsmiR396c regulates *OsGRF4* in rice. (a) Differences in expression pattern of *OsGRF4* between *Nipponbare* and 307R; 1, 2, 3, 4 and 5 indicate a plant with a panicle length of 0, 2, 5, 10 and 15 cm, respectively; (b) *in situ* hybridization analysis of *OsGRF4* and OsmiR396c in rice spikelet hull; scale bar, 200 μm; (c) *OsGRF4* expression pattern during panicle development in *Nipponbare*; (d) OsmiR396c expression pattern during panicle development in *Nipponbare*; (e) RLM‐RACE analysis of the OsmiR396c cleavage sites in the panicle of *Nipponbare* and of 307R; (f–g) sequencing chromatogram of the RT‐PCR products in the heterozygous plants of NIL‐527R/640A (f) and of NIL‐MH63/106A (g), with green arrows indicating mRNA in the NIL forms, whereas red arrows indicate the normal grain forms; values are all shown as means ± SEM.

Growth‐regulating factor genes are known to be substantially regulated by miR396 (Debernardi *et al*., 2014; Hewezi *et al*., 2012; Liu *et al*., 2014; Omidbakhshfard *et al*., 2015; Rodriguez *et al*., 2015). Interestingly, the TC487‐488AA mutation of the large grain rice 307R occurred within the binding site of OsmiR396 (Figure 6a), which might suggest that the OsmiR396 also directly regulated *OsGRF4*. However, the cleaving efficiency of small grains might was different from that of large grains. *In situ* hybridization results showed that *OsGRF4* and OsmiR396c both expressed in rice spikelet hulls (Figure 4b). Detailed quantitative PCR (qPCR) analysis indicated that the expression pattern of *GLW2* was complementary to that of OsmiR396c during panicle and grain development in *Nipponbare* (Figures 4c,d). An RNA ligase‐mediated rapid amplification of cDNA ends (RLM‐RACE) analysis showed that miR396 could directly cleave *OsGRF4* mRNA *in vivo* at one site within the miR396 pairing region in *Nipponbare* (20/20). However, the 2‐bp substitutions of *GLW2* sharply down‐regulated the cleaving efficiency in 307R (2/20) (Figure 4e). We further demonstrated this notion by performing reverse transcription PCR using mRNAs from the heterozygous (NIL‐527R/640A, NIL‐MH63/106A) plants. Sequencing results clearly showed that the mRNAs existed predominantly as the NIL forms (Figures 4f,g), suggesting that cleavage of the NIL‐527R transcripts by OsmiR396 was disrupted. This result was consistent with *OsGRF4* expressed higher in 307R than in *Nipponbare* (Figure 4a).

**Figure 5:**
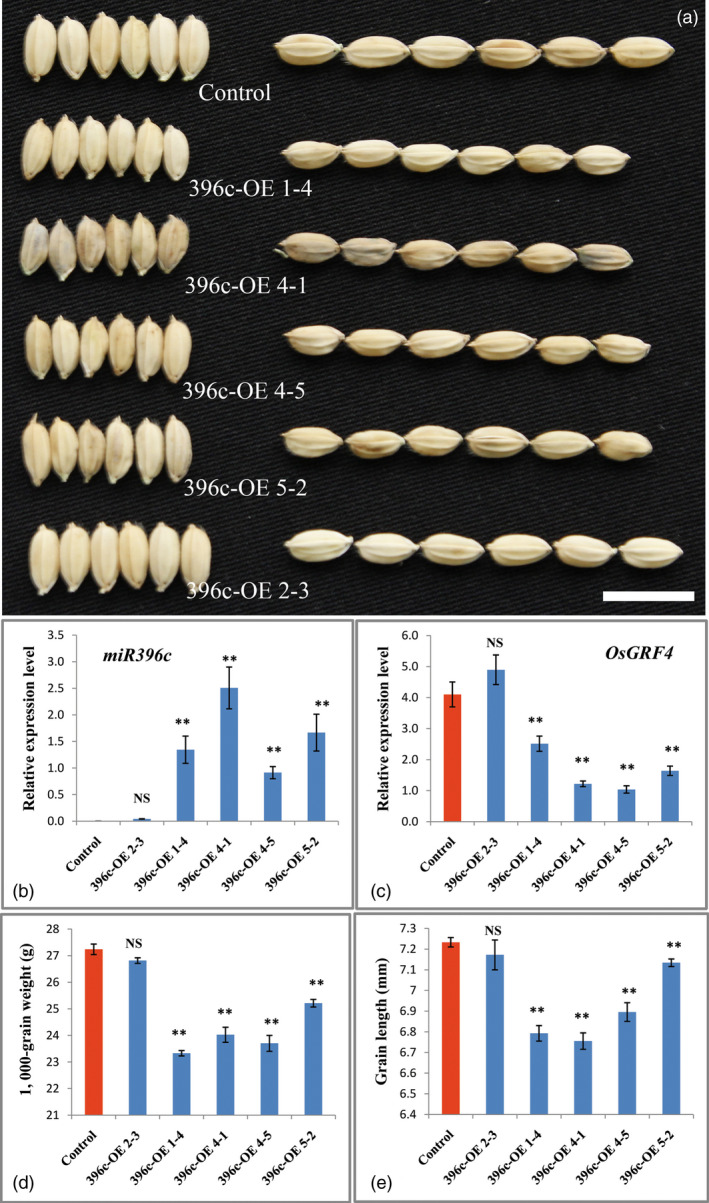
OsmiR396c overexpression decreases rice grain size and weight. (a) Grains of the control and OsmiR396c overexpression plants; scale bar, 10 mm; (b) OsmiR396c expression level of plants in (a); (c) *OsGRF4* expression level of plants in (a); (d) TKW of plants in (a); (e) grain length of plants in (a); values are all shown as means ± SEM (**P < 0.01).

**Figure 6:**
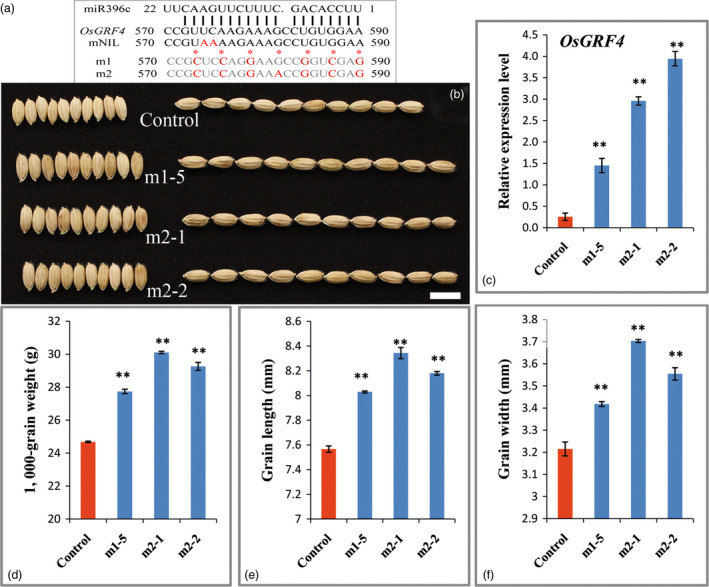
Blocking the target regulation of *OsGRF4* by OsmiR396c leads to a large and heavy grain. (a) Mutant sequence of the two *mOsGRF4* constructs; (b) grains of the control and *mOsGRF4* overexpression plants; Scale bar, 10 mm; (c) *OsGRF4* expression level of plants in (b); (d) TKW of plants in (b); (e) grain length of plants in (b); (f) grain width of plants in (b); values are all shown as means ± SEM (**P < 0.01).

To gain more genetic evidence for the regulation of OsmiR396 on *OsGRF4*, we generated an overexpression construct in which the OsmiR396c was driven by the 2 × 35S promoter and introduced it into *Nipponbare*. Investigations indicated an apparent decrease in the grain size and weight of the transgenic plants (Figures 5a,d,e). The decreased grain size and weight were further confirmed to be a consequence of the substantial decrease in *OsGRF4* transcripts (Figure 5c), which was caused by the overexpression of OsmiR396c (Figure 5b). To confirm whether perturbation of miR396c regulation on *OsGRF4* leads to large grain sizes, two miR396‐resistant variants of *OsGRF4* (*mOsGRF4*) that disrupts the miR396 recognition without changing any amino acid and controlled by the 2 × 35S promoter were introduced into *Nipponbare* (Figure 6a). The transgenic plants exhibited an obviously larger grain size and weight (Figures 6b–f), suggesting that the blocked down‐regulation of *OsGRF4* by miR396c caused the large grain phenotype.

### GLW2 directly interacts with OsGIF1 to regulate grain size

**Figure 7:**
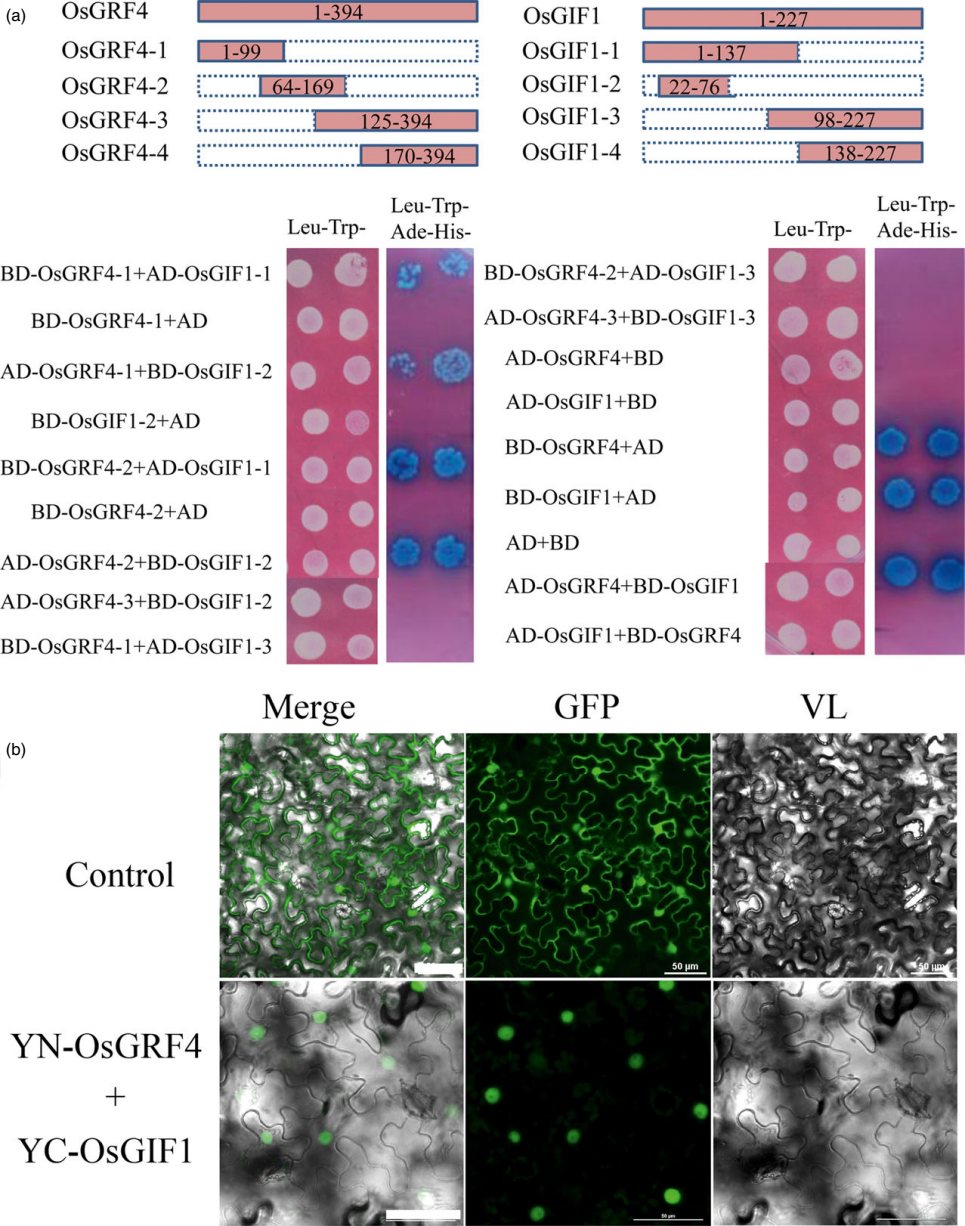
OsGRF4 directly interacts with OsGIF1 in rice. (a) GLW2 interacts with OsGRF4 in yeast. Both GLW2 and OsGIF1 exhibit transcription activation activity; thus, a series of truncations and deletions analyses for both genes were carried out depending on their conserved domains. (b) BiFC analysis of GLW2–OsGIF1 interaction in tobacco leaf. 35S‐YFP was employed as control; scale bar, 50 μm.

Growth‐regulating factors have been widely reported to interact directly with transcription coactivator GIFs (Horiguchi *et al*., 2005; Kim and Kende, 2004; Liu *et al*., 2014). We thus investigated whether GLW2 could interact with rice GIFs using a yeast two‐hybrid system. As shown in Figure 7a, we examined direct interactions between GLW2 and OsGIF1 in yeast cells. Further truncation analysis showed that GLW2 interacted with OsGIF1 at their N‐terminal domains, which is consistent with previous reports on GRFs interacting with partners by the QLQ domain (Horiguchi *et al*., 2005; Kim and Kende, 2004; Liu *et al*., 2014). Bimolecular fluorescence complementation (BiFC) assays further revealed that GLW2 interacted with OsGIF1 in plants (Figure 7b). This result suggests a conserved mechanism between *Arabidopsis* and rice.

**Figure 8:**
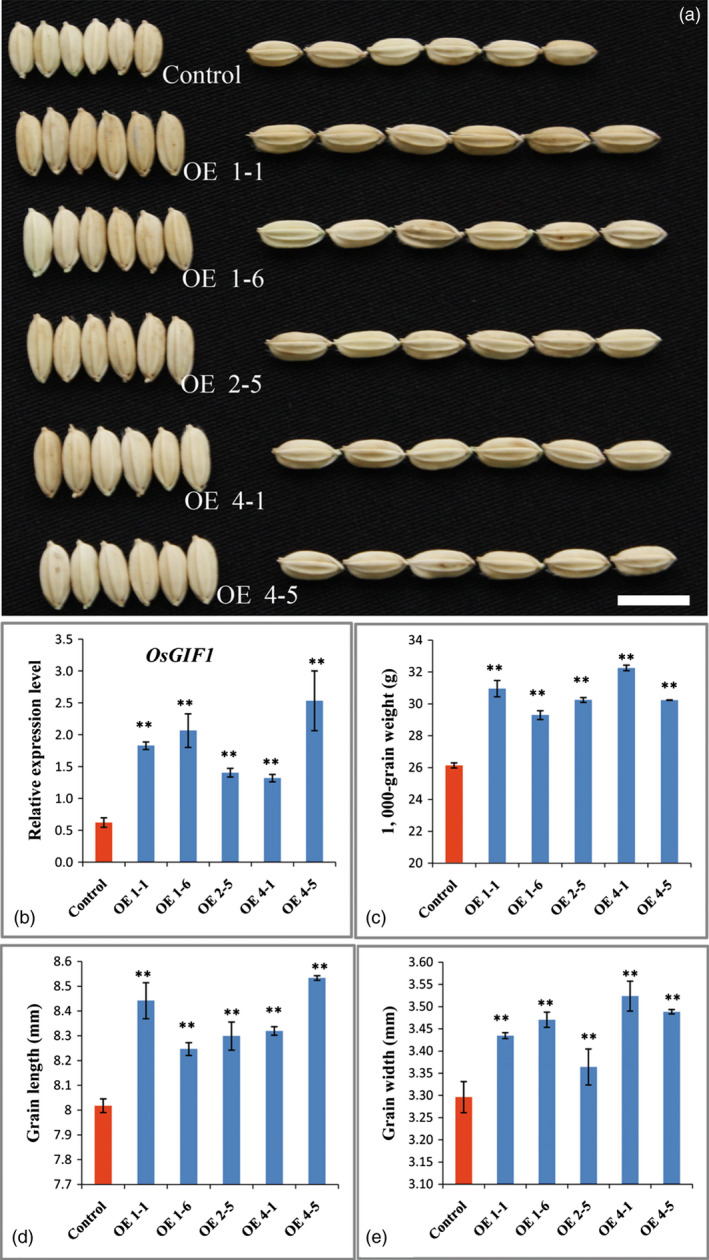
*OsGIF1* overexpression increases rice grain size and weight. (a) Grain comparisons of the control and *OsGIF1* overexpression plants; scale bar, 10 mm; (b) *OsGIF1* expression levels of plants in (a); (c) TGW of plants in (a); (d) grain length of plants in (a); (e) grain width of plants in (a); values are all shown as means ± SEM (**P < 0.01).

To determine whether *OsGIF1* is involved in the seed development process in rice, we then overexpressed *OsGIF1,* which is controlled by the 2 × 35S promoter, in *Nipponbare*. Investigations showed that transgenic plants overexpressing *OsGIF1* (Figure 8b) produced larger and heavier grains than those of *Nipponbare* (Figures 8a,c–e), indicating that *OsGIF1* positively regulated grain growth in rice. Given the above‐mentioned findings, we summarized that GLW2 directly interacted with OsGIFs to manipulate grain size and weight in rice.

### *GLW2* significantly increases hybrid rice yield

Because all the NILs were generated from restorer backgrounds, we can directly use these NILs to evaluate whether *GLW2* can improve the yield of hybrid rice. We crossed those NILs to two currently used cytoplasmic male sterility lines, 106A and 640A, and thus generated several F_1_ hybrid rice lines. Field performance indicated that the *GLW2* locus could significantly increase the plot yield of hybrid rice lines generated from NILs when compared to those generated from recurrent parents (showed a 13.7%–28.0% increase). This increase in plot yield was mainly achieved by increasing the grain length, width, weight and yield per hybrid rice plant (Table S1). Besides, the NIL‐generated hybrid rice was apparently longer in panicle length than that generated from recurrent parents. These data suggest that *GLW2* holds a potential for direct application in high‐yield hybrid rice breeding.

## Discussion

Rice is a highly important food crop because of its role as the main staple food of more than half of the world's population. Grain weight, mainly determined by grain size, is the most important component of rice yield and is generally controlled by QTLs. Although numerous QTLs regulating grain weight have been identified, our understanding of grain size regulation is fragmented. To reveal more genes related to rice grain size and weight, we identified an extra‐large grain rice line designated as 307R from our breeding materials. Genetic analysis indicates that the large grain trait is controlled by an incomplete dominant QTL, suggesting that this locus holds a potential application in rice breeding. As expected, introducing this allele into different backgrounds of rice significantly increases rice grain weight by simultaneously increasing grain length and width. Field performance indicates that *GLW2* locus can also significantly increase the plot yield of hybrid rice lines generated from the NILs by 13.7%–28.0%. Thus, these results suggest that *GLW2* can be exploited in high‐yield hybrid rice breeding.

Map‐based cloning of *GLW2* demonstrated that *GLW2* encodes OsGRF4, a plant‐specific transcription factor. We found that an elevated expression of *OsGRF4* generated enlarged grains, and loss of function resulted in smaller grains. This finding suggests a positive role of *GLW2* in grain size regulation. Very recently, two alleles of *GLW2*,* GS2* (Duan *et al*., 2015) and *GL2* (Che *et al*., 2015) have been reported to positively regulate grain size in rice. *GRFs* have been well documented for their roles in stem and leaf development (Horiguchi *et al*., 2005; Kim and Kende, 2004; Kim *et al*., 2003; van der Knaap *et al*., 2000), root development (Bazin *et al*., 2013; Hewezi *et al*., 2012; Rodriguez *et al*., 2015; Vercruyssen *et al*., 2011), floral organ development (Liang *et al*., 2014; Liu *et al*., 2014), longevity (Debernardi *et al*., 2014) and stress response (Casadevall *et al*., 2013; Hewezi *et al*., 2012; Kim *et al*., 2012). Our results, along with those of other reports (Che *et al*., 2015; Duan *et al*., 2015; Hu *et al*., 2015; Liu *et al*., 2012), demonstrate a new and important role for *GRF* genes in seed size regulation. The study findings suggest *GRF* functions in various plant developmental processes. However, similar to *GS2/GL2*,* GLW2* predominantly promotes cell expansion but also slightly increases cell proliferation in organ size determination, which is different from the case of *Arabidopsis*, in which several *GRFs* have been reported to mainly function in promoting cell proliferation (Horiguchi *et al*., 2005; Omidbakhshfard *et al*., 2015). Further investigations are currently needed to understand the functional distinction of *GRFs* in organ size regulation.

Growth‐regulating factor genes are substantially regulated by miR396 (Debernardi *et al*., 2014; Hewezi *et al*., 2012; Liu *et al*., 2014; Omidbakhshfard *et al*., 2015; Rodriguez *et al*., 2015), which promotes us to survey whether *GLW2* is regulated by OsmiR396. Coincidently, the 2‐bp mutations of the large grain rice 307R occurred within the binding site of OsmiR396. Besides, *GLW2* expression level during grain development in 307R was obviously higher than in *Nipponbare*. Furthermore, *GLW2* and OsmiR396c were co‐expressed in rice spikelet hull and exhibited a complementary pattern during panicle development in *Nipponbare*. These data strongly suggest that *GLW2* may serve as the direct target of OsmiR396. We then confirmed this idea when we noted that the cleavage of *GLW2 in vivo* and the blocking of the target regulation of *OsGRF4* by OsmiR396 generated large and heavy grains. However, eight isoforms (from a–h) exist in the OsmiR396 family; which isoform(s) actually regulate(s) *OsGRF4* is currently unknown (Che *et al*., 2015; Duan *et al*., 2015). The expression of OsmiR396c was consistent with a regulatory role of *OsGRF4*; hence, we further overexpressed the OsmiR396c isoform and found that the overexpression of OsmiR396c significantly decreases the grain size and weight. This finding suggests the regulatory role of the OsmiR396c isoform on *OsGRF4*. However, whether other isoform(s) of this family also play roles in the regulation of *GLW2* remains to be clarified in future study.

Growth‐regulating factors have been widely reported to interact directly with transcription coactivator GIFs (Horiguchi *et al*., 2005; Kim and Kende, 2004; Liu *et al*., 2014). Our results have also demonstrated that GLW2 directly interacts with OsGIF1 both *in vitro* and *in vivo*. Most importantly, overexpression of *OsGIF1* in rice also significantly increases grain size and weight by 13.4%–21.8%, which suggests that *OsGIF1* holds an important role in rice grain size regulation. Overall, our results thus establish that the OsmiR396c‐OsGRF4‐OsGIF1 regulatory module plays roles in grain size determination and enhances rice yield. Considering that OsGRF4 is a plant‐specific transcription factor with intrinsic transcription activation, another important aspect of its function lies in the direct regulation of its downstream target gene. The identification and characterization of the DNA‐binding domain of GLW2 and the GLW2 target gene may offer additional information on the mechanism by which the GRFs contribute to the regulation of grain size and yields in rice.

## Experimental procedures

### Plant materials and growth conditions

The *indica* varieties 307R, IR24, MH63 and 527R and the *japonica* variety of *Nipponbare* were used in this study. Three NILs for *GLW2* were generated by backcrossing 307R with IR24, MH63 and 527R as the recurrent parents, respectively. All plants were planted in the experimental field of the Rice Research Institute, Sichuan Agricultural University, Wenjiang. Phenotypic data were collected at the maturing stage.

### Map‐based cloning of *GLW2*

Normal‐sized F_2_ plants generated from the cross of 307R and IR24 were used for the primary mapping of *GLW2*. For fine mapping, approximately 2500 normal‐sized BC_3_F_2_ plants from the same class were used. Recombinants were self‐crossed and several fixed recombinants were then generated and genotyped with the newly developed markers. All of the new molecular markers used in this process are listed in Table S2.

### Morphological and cellular analyses

The grain length, width and 1000‐grain weight were measured by an automatic seed‐size‐analysing system (SC‐G, Wanshen, Hangzhou, china). For histological analysis, spikelet hulls were placed in Formalin‐acetic acid‐alcohol (FAA) solution (50% alcohol: formalin: glacial acetic acid; 18:1:1) overnight at 4 °C, then dehydrated in a graded ethanol series, followed by substitution using 3‐methylbutyl acetate. The samples were dissected and then observed under a microscope (80I; Nikon, Kanagawa, Japan). An environmental scanning electronic microscope (QUANTA 450; Czech Republic) was employed to observe the outer glume cells.

### Plasmid construction and plant transformation

To verify the *GLW2* function, we firstly generated an overexpression construct by inserting full‐length *GLW2* cDNA from IR24 into the plant binary vector pHB (Mao *et al*., 2005) and introduced the plasmid into *Nipponbare*. We then generated two gRNA constructs, in which the gRNA was driven by the rice U6 promoter and the plant‐optimized *CAS9* was driven by the UBI promoter (Miao *et al*., 2013), and introduced the constructs into NIL‐527R. To verify the miR396c function, a full sequence of pri‐miRNA396c was inserted into pHB and introduced into *Nipponbare*. Two miR396‐resistant variants of *OsGRF4* (*mOsGRF4*) generated by point mutation were inserted into pHB and introduced into *Nipponbare*. We also generated an *OsGIF1* overexpression construct, which we introduced into *Nipponbare* for functional analysis. All constructs were introduced into the *Agrobacterium tumefaciens* strain EHA105. The primer sequences for these constructs are listed in Table S3. Rice transformation was performed in accordance with a previously published method (Hiei *et al*., 1997). To investigate the subcellular localization of GLW2, we constructed a pA7–GLW2–YFP (yellow fluorescent protein) fusion construct whose expression was driven by the CaMV 35S promoter for transiently transforming the rice protoplast. A laser scanning confocal microscope (Nikon A1, Kanagawa, Japan) was used to observe the rice protoplast.

### Transcription activation assay and yeast two‐hybrid assay

The transcription activation assays were conducted with the Matchmaker GAL4 Two‐Hybrid System 2 (Clontech, Dalian, China). The full length and truncations of *GLW2* were fused to the GAL4 DNA‐binding domain. The vectors were then transformed into yeast strain Y2H Gold for transcription activation assays. The full length and truncations of *GLW2* and *OsGIF1* were amplified and subcloned into the pGBKT7 or pGADT7 vectors. The prey and bait plasmids were cotransformed into the yeast strain Y2H Gold and cultured on SD‐Leu‐Trp media for 3 days at 30 °C. Clones were grown on SD‐Ade‐His‐Leu‐Trp medium for 2–3 days at 30 °C for interaction detections.

### BiFC assay

The full‐length cDNA of *GLW2* was cloned into the pXY106 (nYFP) vector, and *OsGIF1* was cloned into the pXY104 (cYFP) vector. These plasmids were co‐expressed in tobacco leaf epidermis cells by *Agrobacterium‐*mediated infiltration. Yellow fluorescent protein was visualized with a confocal scanning microscope (Nikon A1, Kanagawa, Japan) 72 h after infiltration.

### qPCR analysis

Total RNAs from various rice tissues were isolated using the TriPure isolation reagent (Roche, Indianapolis, USA). cDNAs were reverse‐transcribed using the Transcriptor First‐Strand cDNA Synthesis kit (Roche, Indianapolis, USA). For OsmiR396c detection, the microRNAs were isolated using the EASYspin Plant microRNA Kit (Aidlab, Beijing, China). Reverse transcription was conducted using a stem‐loop RT primer ST‐RT1 as described previously (Kramer, 2011). qPCR experiments were carried out in 10 μL reaction mixtures containing 0.3 μL of reverse‐transcribed product, 0.08 mm gene‐specific primers and 5.0 mL of Sso Advanced TM SYBR Green Supermix (Bio‐Rad, California, USA) using a Bio‐Rad CFX96 Real‐Time PCR System (California, USA) in accordance with the manufacturer's instructions.

### RNA *in situ* hybridization

For RNA *in situ* hybridization, spikelet hulls were fixed in FAA solution for 24 h at 4 °C, then dehydrated using a graded ethanol series, followed by a xylene series, and embedded in Paraplast Plus (Sigma‐Aldrich, St Louis, USA) for sections. The sense and antisense RNA probes used in this test are listed in Table S3. Digoxigenin‐labelled RNA probes were prepared using a DIG RNA Labelling Kit (SP6/T7) (Roche, Mannheim, Germany) according to the manufacturer's instructions. A sense probe was used as a negative control.

### RLM‐RACE

RLM‐RACE was conducted in accordance with a GeneRacer kit (Invitrogen, Carlsbad, CA, USA). In general, total RNAs were extracted and the first and second PCRs were performed, with the primers GSP1 and GSP2 (Table S3), respectively. The products from the second PCR were purified by agarose gel electrophoresis and then cloned for sequencing.

## Supporting information


**Figure S1** Yield‐related trait comparisons of NILs and the recurrent parents.
**Figure S2** *GLW2* increases grain size and weight mainly by promoting cell expansion.
**Figure S3** Map‐based cloning of *GLW2*.
**Figure S4** GLW2 is a transcription factor.
**Table S1** Yield performance of the hybrid rice generated by NILs.
**Table S2** Primers used in map‐based cloning.
**Table S3** Primers (or probes) used in molecular cloning, construction and gene expression analysis.
