# Supplementary material for: The OsmiR396c‐OsGRF4‐OsGIF1 regulatory module determines grain size and yield in rice
Source: Plant Biotechnol J. 2016 May 17;14(11):2134–46. doi: 10.1111/pbi.12569 (PMC5095787; doi:10.1111/pbi.12569)
Supplement: Supplementary file 1 — Figure S1 Yield‐related trait comparisons of NILs and the recurrent parents. Figure S2 GLW2 increases grain size and weight mainly by promoting cell expansion. Figure S3 Map‐based cloning of GLW2. Figure S4 GLW2 is a transcription factor. Table S1 Yield performance of the hybrid rice generated by NILs. Table S2 Primers used in map‐based cloning. Table S3 Primers (or probes) used in molecular cloning, construction and gene expression analysis. [file PBI-14-2134-s001.docx]

## Supporting Information

**The OsmiR396c-OsGRF4-OsGIF1 regulatory module determines grain size and yield in Rice**

Shuangcheng Li^1,2,*^ Fengyan Gao^1, *^ Kailong Xie^1^ Xiuhong Zeng^1^ Ye Cao^1^ Jing Zeng^1^ Zhongshan He^1^ Yun Ren^1^ Wenbo Li^1^ Qiming Deng^1,2^ Shiquan Wang^1,2^ Aiping Zheng^1,2^ Jun Zhu^1,2^ Huainian Liu^1,2^ Lingxia Wang^1,2^ Ping Li^1,2,#^

**
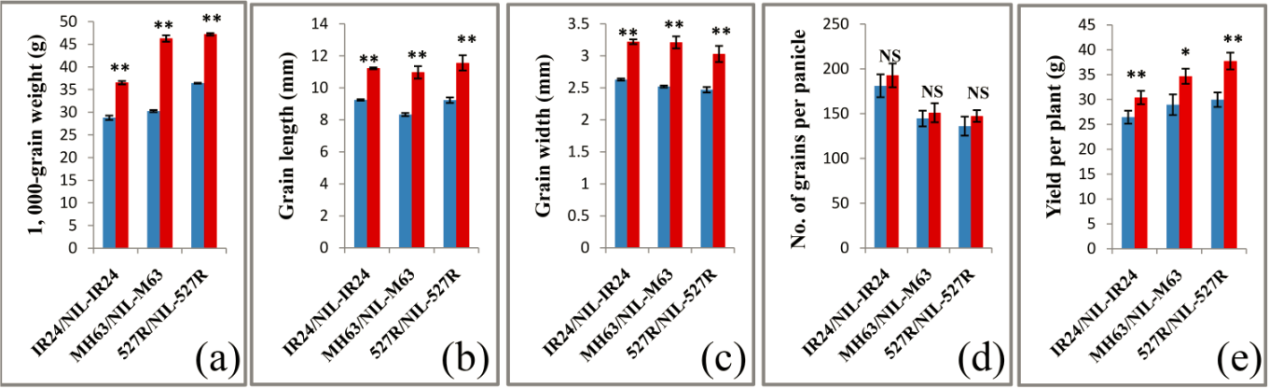
**

**Figure S1 Yield-related trait comparisons of NILs and the recurrent parents**

(a) TGW;

(b) Grain length;

(c) Grain width;

(d) Grain number;

(e) Yield per plant.

Values are all shown as means ± s.e.m.


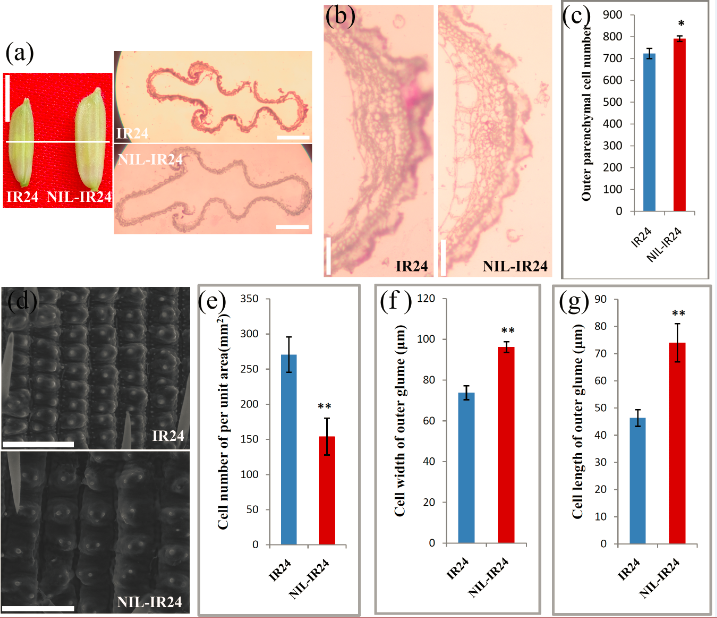


**Figure S2. *GLW2* increases grain size and weight mainly by promoting cell expansion**

(a) Cross sections of spikelet hulls of IR24 and NIL-IR24. The white line indicates the position of the cross section. Scale bar, 5 mm in the left and 500 µm in the right.

(b) Magnified view of the cross section in (a); Scale bar, 100 µm.

(c) Comparison analysis of the cell number in the outer parenchymal layer.

(d) Scanning electron microscope analysis of the outer surfaces of glumes in IR24 and NIL-IR24; Scale bar, 200 µm.

(e–g) Comparison analysis of cell number per unit area (e), cell width (f) and length (g) in outer glumes.

Values are all shown as means ± s.e.m.


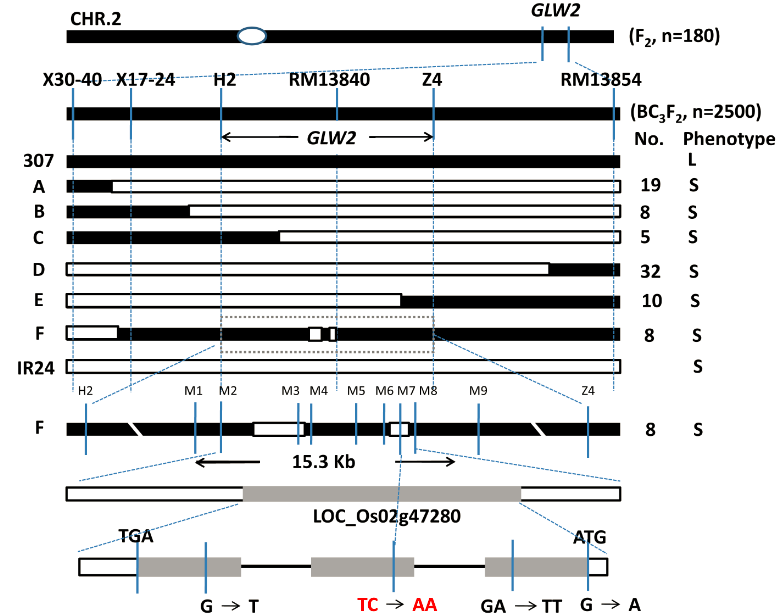


**Figure S3. Map-based cloning of *GLW2***

*GLW2* was first mapped to chromosome 2 and was further narrowed down to a 160 kb interval between the markers H2 and Z4. Finally, the *GLW2* locus was limited to a 15.3 kb interval between the markers M2 and M8 by fine genotyping of eight fixed recombinants (F group). The coding regions of the candidate gene *LOC_Os02g47280* have four polymorphisms between *Nipponbare* and 307R; however, only one polymorphism (TC487-488AA) is conserved between IR24/MH63/527R and their NILs. A–F indicates different groups of fixed recombinants classified by fine genotyping with newly developed markers (M1–9), numbers at the right indicates the numbers of fixed recombinants in each group, L indicates a large grain phenotype, whereas S indicates a small one.


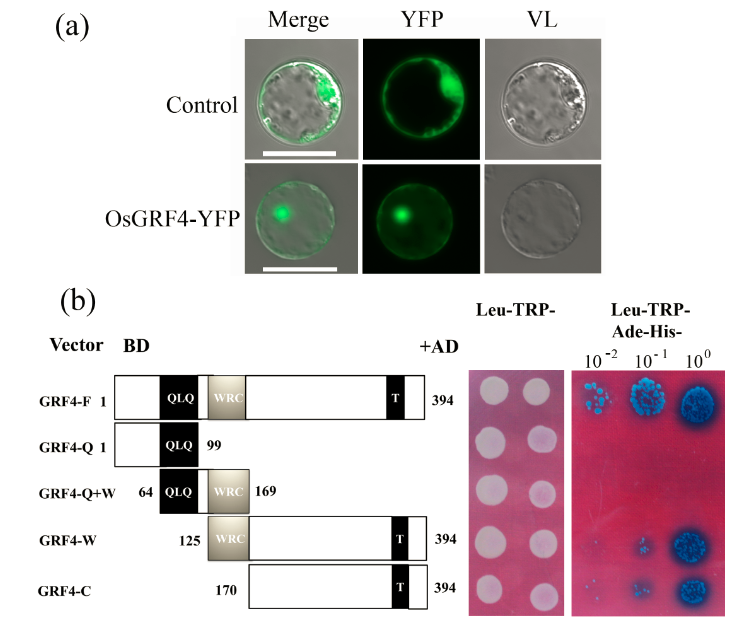


**Figure S4 GLW2 is a transcription factor**

(a) Subcellular localisation of GLW2 with 35S-YFP used as control; Scale bar, 25 µm;

(b) GLW2 transcription activation analysis in yeast. The full-length, N-terminal deletion, C-terminal deletion and NC-deletion cDNAs of GLW2 were analysed in the yeast cell.

**Table S1. Yield performance of the hybrid rice generated by NILs**

| Trait | 640A/MH63 | 640A/NIL-MH63 | 106A/MH63 | 106A/NIL-MH63 | 640A/527R | 640A/NIL-527R |
| --- | --- | --- | --- | --- | --- | --- |
| Grain length (mm) | 9.28±0.03 | 10.68±0.10****** | 10.72±0.089 | 12.43±0.088****** | 9.88±0.07 | 11.29±0.07****** |
| Grain width (mm) | 2.58±0.05 | 2.90±0.04****** | 2.57±0.015 | 2.86±0.011****** | 2.62±0.05 | 2.93±0.01****** |
| 1,000-grain weight (g) | 27.86±0.25 | 36.49±0.07****** | 31.63±0.043 | 47.12±1.11****** | 30.37±0.12 | 38.87±0.29****** |
| Yield per plant (g) | 40.40±4.13 | 55.77±3.29***** | 37.03±3.430 | 53.49±2.11***** | 39.40±2.829 | 52.34±3.40***** |
| Yield per plot (g/m^2^) | 898.00±38.68 | 1149.50±11.20****** | 1391.1±25.33 | 1582.20±47.29***** | 931.30±54.13 | 1097.50±12.99***** |
| Panicle length (cm) | 24.03±0.14 | 27.73±0.34****** | 28.50±0.29 | 34.17±0.44****** | 25.28±0.06 | 28.50±0.38****** |
| Tiller number | 20±1.201 | 16±0.58***** | 13±1.20 | 11±0.58 | 12±1.12 | 12±2.03 |
| Plant height (cm) | 96.87±1.94 | 109.93±0.23****** | 120.33±0.33 | 125.33±0.33****** | 104.83±2.42 | 106.53±1.89 |

Values are all shown as means ± s.e.m.

**Table S2. Primers used in map-based cloning**

| Primer name | Sequence (5’-3’) | Purpose |
| --- | --- | --- |
| X30-40-F | CAGGGCTCATACCTTTGTTC | Mapping |
| X30-40-R | AGATATGGATGCTGTTTCTC | Mapping |
| X17-24-F | GAGACTTGGAAGGAATCTTG | Mapping |
| X17-24-R | TGGACACTGTTCATTCAAAC | Mapping |
| H2-F | AACTATTAGCGTGTGTTC | Mapping |
| H2-R | CAAGAGTGGTGTCGTGCC | Mapping |
| RM13840-F | TGCTTTGCTTCGCTCGCT | Mapping |
| RM13840-R | GAGCGCGCCTTCTCGTGT | Mapping |
| Z4-F | CTCGCAGAAACCTTTCCCGTA | Mapping |
| Z4-R | GGTATCATCTAGTGCGTGCG | Mapping |
| RM13854-F | CGTCAGTAATGGCGGATCTTGC | Mapping |
| RM13854-R | CTTCGCTATCACCGAAGGAACC | Mapping |
| M1-F | AGTTGTGCTTAAAGTACTGT | Mapping |
| M1-R | TCCCACAAATTCACTCGGAA | Mapping |
| M2-F | TGTAAAGGCAATCATTAGGA | Mapping |
| M2-R | GACGCGTTTGAATCATAACA | Mapping |
| M3-F | AGAGTACATACAGGAATCCA | Mapping |
| M3-R | TCAGCACCCTTTACTTCTAT | Mapping |
| M4-F | ACAGCGTTTGTCGAGACTTCT | Mapping |
| M4-R | AGCCGGTGTTGCTCTATATT | Mapping |
| M5-F | AACAAACCGGATGGCCCAA | Mapping |
| M5-R | ACCGTTTGATTGAGCCGCAT | Mapping |
| M6-F | ATCGCAGAGCAAGACAGCAA | Mapping |
| M6-R | TGATGCTCCAAGTCAACTCT | Mapping |
| M7-F | TGATTCAAGACAGTGCTAAACAAGT | Mapping |
| M7-R | TGGATCGGATATGGACTTCTCA | Mapping |
| M8-F | TGCTTTGCTTCGCTCGCT | Mapping |
| M8-R | GAGCGCGCCTTCTCGTGT | Mapping |
| M9-F | AGCGTACTACCTCTATTTCA | Mapping |
| M9-R | ACAGTCTGAGTTGGCACTGT | Mapping |
| W162-F | TTCTTCTGGTGTGTGCCTTG | Mapping |
| W162-R | GGGGAAGGAGGATGCTAAAC | Mapping |
| W189-F | GTGTGTACGTAGGATCGGAG | Mapping |
| W189-R | TGCTACTCCTAGCTGCTACC | Mapping |
| W190-F | ATACATGCATACTCCGATCC | Mapping |
| W190-R | TCGAGTTGTGAAAGATAGGG | Mapping |
| W193-F | AGAAACCAAAGATGGGAGGG | Mapping |
| W193-R | CTAGCCAGCTCTCCCTTTTG | Mapping |
| RM5305-F | TGCTGCATCTCCATATCACAAGG | Mapping |
| RM5305-R | TCCCTCTCACTTCACACAGATGG | Mapping |

**Table S3. Primers (or probes) used in molecular cloning, construction and gene expression analysis**

| Primer name | | Sequence (5’-3’) | Purpose |
| --- | --- | --- | --- |
| OsGRF4C-HA-F | GCCGGATCCATGGCGATGCCGTATGCCTCC | Nipp-cDNA,Transgenic construct |  |
| OsGRF4C-HA-R | GGACTAGTGTCACCATTAGTTGATCGAG | Nipp-cDNA,Transgenic construct |  |
| OsGIF1C-F | GCCGGATCCATGCAGCAGCAACACCTG | GIF1-cDNA,Transgenic construct |  |
| OsGIF1C-R | GGACTAGTGCTGCCTTCCTCCTCGGTG | GIF1-cDNA,Transgenic construct |  |
| cz396cF | CACCAGTCTCTCTCTCAAGCTT GCCATAAAGCTCTTCGTCCT | miR396 Overexpression, Transgenic construct |  |
| cz396cR | TCTAGAGGATCAATTCGAGCTC TGCCTGTCTCATGGCATTGC | miR396 Overexpression, Transgenic construct |  |
| OsGRF4-YFP-F | GCCGGATCCATGGCGATGCCGTATGCCT | YFP, Subcellular localization |  |
| OsGRF4-YFP-R | GGACTAGTGTCACCATTAGTTGATCGAG | YFP, Subcellular localization |  |
| OsGRF4-1-99-R | GCCGGATCCGCGGCGGATGGGGAGCA | Y2H construct |  |
| OsGRF4-64-169-F | CGGAATTCCCGTTCACCGCGGCGCAGTA | Y2H construct |  |
| OsGRF4-64-169-R | GCCGGATCCCGTTTCCACAGGCTTTCT | Y2H construct |  |
| OsGRF4-125-390-F | CGGAATTCGACCCAGAGCCAGGGCGGTG | Y2H construct |  |
| OsGRF4-125-390-R | GCCGGATCCTCAGTCACCATTAGTTGAT | Y2H construct |  |
| OsGRF4-170-390-F | CGGAATTCCAGCTGGTCGCCCAGTCCCA | Y2H construct |  |
| OsGRF4-170-390-R | GCCGGATCCTCAGTCACCATTAGTTGAT | Y2H construct |  |
| OsGIF1-1-137-F | CGGAATTCATGCAGCAGCAACACCTGAT | Y2H construct |  |
| OsGIF1-1-137-R | GCCGGATCCCGCCGCCTGCTGCTGCTGC | Y2H construct |  |
| OsGIF1-22-76-F | CGGAATTCACCGTCACCACTGATCTCA | Y2H construct |  |
| OsGIF1-22-76-R | GCCGGATCCCGGCTGGCTGTCGGCGAT | Y2H construct |  |
| OsGIF1-98-196-F | CGGAATTCATGCCGCAGCAGTCGGCGC | Y2H construct |  |
| OsGIF1-98-196-R | GCCGGATCCCTAGCTGCCTTCCTCCTC | Y2H construct |  |
| OsGIF1-138-196-F | CGGAATTCGCGGCGCACGGGCAGCTGGGC | Y2H construct |  |
| OsGIF1-138-196-R | CGGAATTCGCGGCGCACGGGCAGCTGGGC | Y2H construct |  |
| 104-OsGRF4C-F | GCCGGATCCATGGCGATGCCGTATGCCTC | BiFC construct |  |
| 104-OsGRF4C-R | GCTTCTAGAGTCACCATTAGTTGATCGAG | BiFC construct |  |
| 106-OsGIF1C-F | GCCGGATCCATGCAGCAGCAACACCTGA | BiFC construct |  |
| 106-OsGIF1C-R | GCTTCTAGAGCTGCCTTCCTCCTCGGT | BiFC construct |  |
| qOsGRF4-F | ATGCCCAGCTCATTTGGCT | qPCR |  |
| qOsGRF4-R | CCGCCAAAGATCTTGTGCCA | qPCR |  |
| qOsGIF1-F | TGCTTCCCCTACCACCGTC | qPCR |  |
| qOsGIF1-R | CGAGCGCACTCTTCCACCT | qPCR |  |
| SL-RT1 | GTCGTACCAGTGCAGGGTCCGAGGTATTCGC  ACTGGATACGACAAGTTC | Stem-Loop |  |
| Os396C-F | GCGCCTTTCCACAGCTTTCTTGAAC | qPCR |  |
| Os396C-R | CCAGTGCAGGGTCCGAGGTA | qPCR |  |
| GSP1 | GCCGCCGAGAAGTCAGAGGATGCC | 5′RACE |  |
| GSP2 | GGAAATGGCGAGTTCTGAGATGGCA | 5′RACE |  |
| UBi-F | ACCCTGGCTGACTACAACATC | qPCR |  |
| UBi-R | AGTTGACAGCCCTAGGGTG | qPCR |  |
| U6 F | GGGACATCCGATAAAATTGGAA | qPCR |  |
| U6 R | CGATTTGTGCGTGTCATCCTT | qPCR |  |
| SmiR396C | TTCCACAGCTTTCTTGAACTT | RNA *in situ* hybridization |  |
| ASmiR396C | AAGTTCAAGAAAGCTGTGGAA | RNA *in situ* hybridization |  |
| SOsGRF4 | TATGCAGCTGTTGGTGGTGGAACAGGCAAAGATCTCAGGTATACTGCTTAT  GGCACAAGATCTTTGGCGGATGAGCAGAGTCAACTCATTACTGAAGCTATCAA | RNA *in situ* hybridization |  |
| ASOsGRF4 | TTGATAGCTTCAGTAATGAGTTGACTCTGCTCATCCGCCAAAGATCTTGTGCC  ATAAGCAGTATACCTGAGATCTTTGCCTGTTCCACCACCAACAGCTGCATA | RNA *in situ* hybridization |  |
